# The Role of Orientation of Surface Bound Dihydropyrrol-2-ones (DHP) on Biological Activity

**DOI:** 10.3390/molecules24142676

**Published:** 2019-07-23

**Authors:** Aditi Taunk, Renxun Chen, George Iskander, Kitty K. K. Ho, Basmah Almohaywi, David StClair Black, Mark D. P. Willcox, Naresh Kumar

**Affiliations:** 1 School of Chemistry, University of New South Wales, Sydney 2052, Australia; 2 School of Optometry and Vision Science, University of New South Wales, Sydney 2052, Australia

**Keywords:** quorum sensing, surface attachment, *Pseudomonas*, *Staphylococcus*, antimicrobial, biomaterials

## Abstract

Quorum sensing (QS) signaling system is important for bacterial growth, adhesion, and biofilm formation resulting in numerous infectious diseases. Dihydropyrrol-2-ones (DHPs) represent a novel class of antimicrobial agents that inhibit QS, and are less prone to develop bacterial resistance due to their non-growth inhibition mechanism of action which does not cause survival pressure on bacteria. DHPs can prevent bacterial colonization and quorum sensing when covalently bound to substrates. In this study, the role of orientation of DHP compounds was investigated after covalent attachment by 1-ethyl-3-(3-dimethylaminopropyl)carbodiimide (EDC)/*N*-hydroxysuccinimide (NHS) coupling reaction to amine-functionalized glass surfaces via various positions of the DHP scaffold. The functionalized glass surfaces were characterized by X-ray photoelectron spectroscopy (XPS) and contact angle measurements and tested for their in vitro biological activity against *S. aureus* and *P. aeruginosa*. DHPs attached via the N-1 position resulted in the highest antibacterial activities against *S. aureus*, while no difference was observed for DHPs attached either via the N-1 position or the C-4 phenyl ring against *P. aeruginosa.*

## 1. Introduction

According to the World Health Organization (WHO), infectious diseases are the second leading cause of death and are responsible for approximately 15 million deaths every year worldwide [1]. Bacteria are responsible for about 90% of all infections [2]. According to estimates from the USA Centers for Disease Control and Prevention, almost 2 million people are infected with bacteria that are resistant to antibiotics, and at least 23,000 people die as a direct result of these infections each year in the USA [3]. Also, recent reports suggest that by 2050 drug-resistant infections will be the leading cause of deaths in the world with the death rate going up to 10 million each year [4,5].

The insertion of indwelling or implanted foreign polymer bodies such as cardiovascular devices [6], dental [7,8], orthopaedic [9], cochlear implants [10,11], catheters [12,13,14], contact lenses [15], and many more have become an indispensable part of modern medical care. Many are life saving devices that are responsible for significantly improving the quality of life and also increasing the life expectancy of patients [16]. However, the insertion or implantation of medical devices has been associated with a risk of microbial infections [17,18].

A promising strategy to prevent infections of biomedical devices is to coat the material’s surface with a suitable antibacterial agent. Compared to conventional antibiotics, quorum sensing (QS) inhibitors such as dihydropyrrolones (DHPs) are excellent coating agents for medical implants since they are potentially less liable to induce the development of antibacterial resistance and have broad spectrum antibacterial activity with low cytotoxicity [19,20,21,22,23].

The surface concentration of covalently-attached DHPs can have a large impact on the activity of coated surfaces, with higher amounts of specifically-bound DHPs leading to increased biofilm inhibition [22]. However, it was observed in a previous study that the activity of modified surfaces was not greatly affected by the concentration of DHPs which were grafted by non-specific attachment [24]. These surfaces were equally effective in preventing bacterial colonization at low concentrations against two common pathogens, *Staphylococcus aureus* and *Pseudomonas aeruginosa*. This difference in the efficacy of DHPs could be due to changes in the orientation of DHP when immobilized on the surface via non-specific attachment strategy, randomly exposing the more active part of molecule to the bacteria.

For the development of efficient and long-lasting antibacterial devices and implants, it is important to understand the effect of the orientation of the attached active molecule on its activity. To date, no systematic study has been carried out to investigate the effect of different orientations of DHP on antibacterial activity. In the current study, potent DHP compounds that have been reported in previous studies were covalently linked to amine-functionalized glass surface via various positions on the DHP scaffold by EDC/NHS coupling reaction. The functionalized glass surfaces were characterized by XPS and contact angle measurements and tested for their antimicrobial activity against *S. aureus* and *P. aeruginosa*.

## 2. Results

### 2.1. Synthesis of DHP Analogues

**Figure 1:**
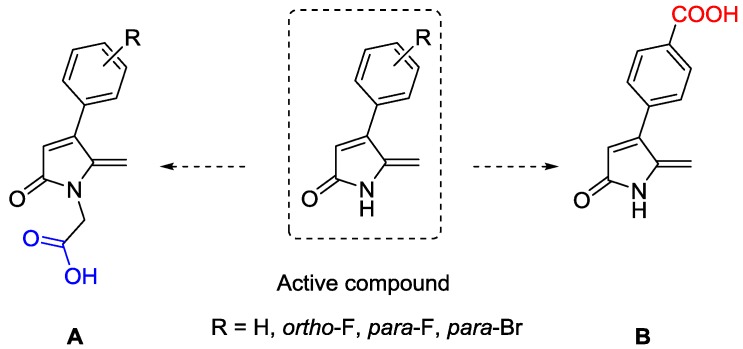
Functionalization of active dihydropyrrolones (DHP) with carboxylic acid group at N-1 position (**A**), and C-4 phenyl ring (**B**).

A series of DHP analogues having a similar structural skeleton were functionalized with a free carboxylic acid group at the N-1 position (Figure 1A) with different substituents at the phenyl C-4 position according to a modification of a method developed by Kumar and Iskander [20]. This generated a site of attachment at the *N*-atom of the lactam ring with minimal change to the molecular structure of the DHP. Further, a carboxylic acid group was introduced at the *para* position of C-4 phenyl ring of DHP to provide an alternative attachment site on the other side of the molecule. (Figure 1B). Previously, para-bromo and fluorine substitutions on the phenyl ring were shown to be potent antimicrobial DHP compounds [25]. Hence, the four acid-functionalized DHP compounds used in this study were chosen with these substitutions on the phenyl ring.

**Scheme 1:**
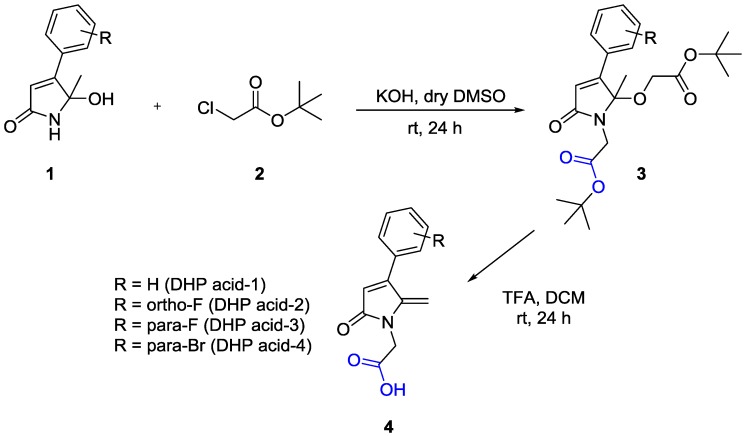
Synthesis of acid derivatives of DHPs (DHP acids 1–4).

The acid-functionalized DHP analogues (DHP acid 1–4) were synthesized via a *tert*-butyl acetate DHP intermediate which was in turn obtained by reacting various 5-hydroxy-5-methyl-4-aryl-1,5-dihydro-2*H*-pyrrol-2-ones **1** with *tert*-butyl chloroacetate **2** in the presence of potassium hydroxide (Scheme 1). The proton NMR spectra of the intermediates, *tert*-butyl-2-(5-(2-(*tert*-butoxy)-2-oxoethoxy)-5-methyl-2-oxo-4-aryl-1,5-dihydro-2H-pyrrol-1-yl)acetate **3**, confirmed the presence of two *tert*-butyl acetate groups in each compound by the appearance of a singlet at 1.46 ppm for the CH_3_ groups. In the next step the intermediate compounds were treated with trifluoroacetic acid, which facilitated cleavage of ether at C-5 resulting in removal of the ester group followed by dehydration to form the characteristic C-5 vinylic double bond. At the same time the N-1 ester group underwent hydrolysis in the presence of trifluoroacetic acid to convert it to carboxylic acid. The disappearance of the peaks for the C-5 acetate group and N-1 *tert*-butyl group in the NMR spectrum indicated completion of the reaction. The crude product was washed with saturated sodium bicarbonate and dried under vacuum to give the pure product of 2-(5-methylene-2-oxo-4-aryl-1,5-dihydro-2*H*-pyrrol-1-yl)acetic acid **4** (DHP acids 1–4).

**Scheme 2:**
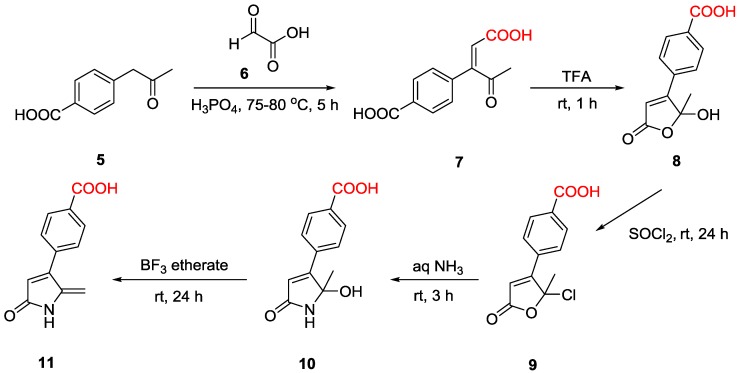
Synthetic route for *p*-acid DHP.

The *p*-acid DHP compound was synthesized through a series of steps as shown in Scheme 2. 4-(2-Oxopropyl)benzoic acid **5** was reacted with glyoxylic acid **6** at 75–80°C for 5 h to generate the di-acid compound in 50% yield after flash column chromatography purification. The identity of the di-acid product, (*Z*)-4-(1-carboxy-3-oxobut-1-en-2-yl)benzoic acid **7**, was confirmed by proton NMR spectroscopy. Cyclization of the di-acid compound to form the corresponding furanone compound **8** with a hydroxyl group at C-5 was accomplished in the next step by using trifluoroacetic acid. The reaction was monitored by TLC which confirmed formation of the hydroxyl furanone after 1 h.

The next step involved a lactone to lactam ring conversion which was initially attempted by reaction of the hydroxyl furanone with aqueous NH_3_. However, this was unsuccessful due to the formation of multiple unexpected products. Instead, the C-5 hydroxyl group was replaced with chlorine by using thionyl chloride as the chlorinating agent. To avoid concomitant chlorination of the carboxylic acid group, the reaction was carried out at room temperature. The reaction was continuously monitored by TLC which indicated the reaction was complete after 24 h. The 5-chlorofuranone compound **9** successfully underwent the lactone to lactam conversion with aqueous ammonia giving the desired DHP product, 5-hydroxy-5-methyl-4-(4-carboxyphenyl) -1,5-dihydro-2*H*-pyrrol-2-one **10**, after 3 h. The formation of the DHP product was confirmed by its proton NMR spectrum in DMSO, which exhibited a characteristic broad peak at 8.5 ppm representing the –NH group of the lactam ring.

The final step involved dehydration of the hydroxyl DHP **10** to regenerate the methylene group at the C-5 position. Dehydration was carried out by using borontrifluoride etherate as the dehydrating agent. The proton NMR analysis of the product showed the presence of two singlet peaks at 5.19 ppm and 6.42 ppm corresponding to the two protons of the C-5 double bond, indicating successful dehydration to form 4-(4-carboxy phenyl)-5-methyelene-1,5-dihydro-2*H*-pyrrol-2-one **11** (*p*-acid DHP)**.**

**Figure 2:**
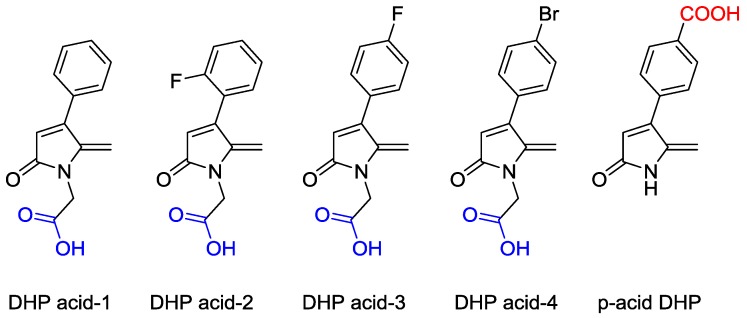
Chemical structures of DHP derivatives synthesized.

Overall, four DHPs with carboxylic acid groups at the N-1 position of the lactam ring (DHP acids 1–4) and one DHP analogue with a carboxylic group at the C-4 pendant phenyl ring (*p*-acid DHP) were synthesized (Figure 2).

### 2.2. Surface Characterization by X-ray Photoelectron Spectroscopy (XPS)

**Scheme 3:**
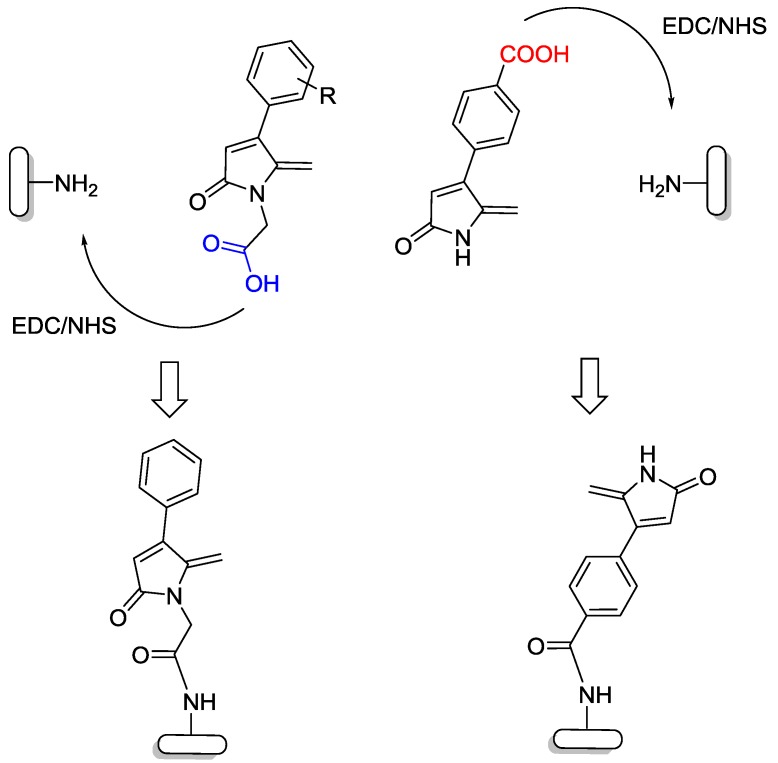
Specific covalent attachment of DHPs on amine-functionalized surfaces using the 1-ethyl-3-(3-dimethylaminopropyl)carbodiimide (EDC)/*N*-hydroxysuccinimide (NHS) coupling reaction.

The resulting acid-DHP derivatives were grafted onto (3-aminopropyl)triethoxysilane (APTES)-functionalized glass substrates by the 1-ethyl-3-(3-dimethylaminopropyl)carbodiimide (EDC)/*N*-hydroxysuccinimide (NHS) coupling reaction (Scheme 3). XPS elemental analysis was carried out to determine the surface composition after every modification step. The elemental composition of the glass surfaces is shown in Table 1.

The changes in the carbon, nitrogen, and halogen composition on the glass surface before and after attachment of APTES and DHPs indicated successful surface modification (Table 1). The carbon and nitrogen concentration increased drastically from 6.6% and 0.6% to 45.4% and 8.1%, respectively, after functionalization of surface by APTES [24,26]. The subsequent attachment of DHP acids 1–4 and *p*-acid DHP further increased carbon content by 1.8%–6.3% and 4.8% respectively compared to the APTES control. Similarly, the nitrogen concentration also increased by 0.7%–2.7% after reaction with DHP acids and by 1.1% for *p*-acid DHP. Furthermore, the halogens detected in the XPS analysis confirmed the attachment of halogenated DHP acids 2–3 on the APTES glass surface. As indicated by the halogen content, the highest attachment efficiency was displayed by DHP acid-4 (0.91% Br) which was approximately 2–5 times higher than the fluorine substituted DHPs. This was followed by DHP acid-2 (0.4% *ortho*-F) which had roughly twice the amount of fluorine as DHP acid-3 (0.17% *para*-F). In absence of halogen, the surface coverage of DHP acid-1 and *p*-acid DHP was determined by the carbon and nitrogen values which indicate both the non-halogenated DHPs have similar coverage on the APTES glass surface.

The curve fitting results for C 1s and N 1s regions and proposed assignments based on chemical shifts are shown in Table 2. The C 1s spectrum for APTES glass showed three carbon species assigned to an aliphatic carbon at 284.9 eV, C-N at 285.9 eV and C=O at 287.9 eV. For the DHP-treated surfaces, a new peak emerged at ~288.6 eV corresponding to the amide bond (N-C=O), indicating successful reaction of DHPs with the APTES surface.

The N 1s high resolution scan for all the glass samples showed peaks at around 399.5 eV and 401.5 eV which were assigned to an amine bond and NH_3_^+^/tertiary nitrogen respectively (Table 2). For the DHP glass surfaces, the peak at ~400 eV was attributed to the nitrogen from the amide bond (N-C=O), which is an indication of surface modification by the DHP compounds. The ratio of the nitrogen species for all the glass surfaces changed after DHP modification, notably with an increase in peak area for the amide bond.

### 2.3. Contact Angle Measurements

Static water contact angle measurements were employed to determine the changes in hydrophobicity of the surface which indicates changes in surface chemical composition after every step. The hydrophobicity of materials is a useful parameter that is correlated with cell-biomaterial interfacial interactions [27,28]. The contact angle of the uncoated blank glass surface was 19° (Table 3). After modification with APTES, a significant increase in surface hydrophobicity was observed, with a contact angle value of 72°. The contact angle values remained approximately the same (68–75°) after subsequent attachment of DHPs due to the relatively hydrophobic nature of the DHPs.

### 2.4. Antibacterial Activity

**Figure 3:**
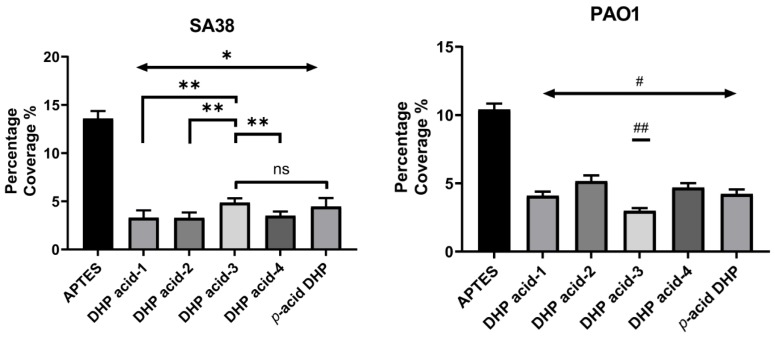
Percentage surface coverage of live and dead bacteria (mean ± standard deviation of mean) for *S. aureus* SA38 (Left) and *P. aeruginosa PAO1* (Right). For *S. aureus SA38*: * Indicates *p* < 0.001 compared to control; ** indicates *p* < 0.05 compared to DHP acid-3 modified glass surfaces. For *P. aeruginosa* PAO1: ^#^ Indicates *p* < 0.001 compared to control; ^##^ indicates *p* < 0.05 to all other groups.

**Figure 4:**
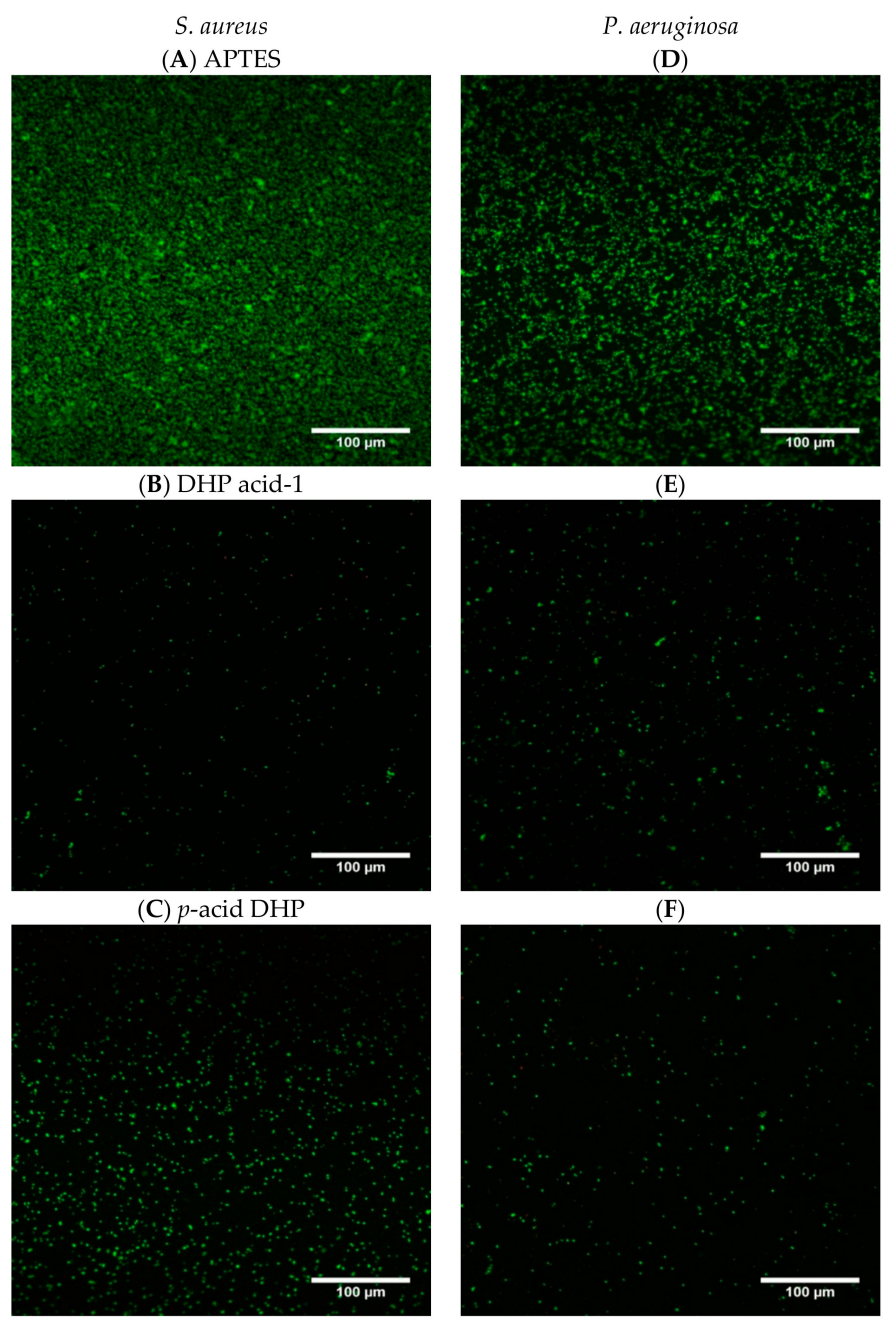
Confocal microscopic images of *S. aureus* and *P. aeruginosa* adhered to APTES process control (**A**,**D**), DHP acid-1 surfaces (**B**,**E**) and *p*-acid DHP surfaces (**C**,**F**). Live bacterial cells stained green and dead bacteria stained red. Magnification 200×. Scale bar = 100 µm.

The adhesion of *S. aureus* and *P. aeruginosa* to the modified surfaces was investigated using fluorescence microscopy by staining the surfaces using the BacLight Live/Dead Bacterial Viability kit. The images were analyzed to determine the relative proportion of live and dead bacteria (stained green or red respectively) on each surface and the results for *S. aureus* SA38 and *P. aeruginosa* PA01 are shown in Figure 3 and Figure 4, respectively.

The APTES control surfaces showed extensive colonization of *S. aureus*, with bacterial coverage of more than 13% (Figure 3). The untreated blank glass surface (data not shown) displayed similar levels of adhesion, suggesting that APTES modification does not have a significant effect on bacterial adhesion. Encouragingly, the DHP-coated glass surfaces displayed significantly lower bacterial coverage compared to the ATPS-coated glass surface. Specifically, the DHP acids 1–4 and p-acid DHP displayed reductions in surface coverage by 64.2%–75.8% and 68.2% respectively compared to APTES (*p* < 0.001). Among the various DHP acids, surfaces modified with DHP acid-1, DHP acid-2, and DHP acid-4 were the most active against *S. aureus* adhesion and statistically different to DHP acid-3 and *p*-acid DHP (*p* < 0.05). DHP acid-1 (75.8 ± 0.8%), DHP acid-2 (75.8 ± 0.5%), and DHP acid-4 (74.2 ± 0.8%) showed similar efficacies at reducing bacterial coverage, and all three performed better than DHP acid-3 (64.2 ± 0.4%) and *p*-acid DHP which reduced 68.2 ± 0.4% of *S. aureus* adhesion.

There was no significant increase in the proportion of dead (red-staining) cells for all modified samples indicating the surfaces inhibit bacterial attachment rather than killing the bacteria (not shown). This may result in a low selective pressure for development of resistance to DHPs [29,30]. Representative micrographs of *S. aureus* adhesion on APTES, DHP acid-1, *p*-acid DHP, surfaces are shown in Figure 4.

The image analysis results for *P. aeruginosa* showed high bacterial coverage on APTES control surfaces (>10%; Figure 3) and blank glass surfaces (data not shown). Significant reductions in adherent bacterial cells was observed for all the DHP-modified glass surfaces compared to the APTES control (50.4%–71.3% reduction for DHP acids 1–4 and 60.1% for *p*-acid DHP; *p* < 0.001). DHP acid-3 (71.3 ± 0.2%) showed the highest bacterial reduction compared to the other DHP acids (*p* < 0.05). There were no significant differences between the DHP acid-1 and *p*-acid DHP (60.1 ± 0.3%) modified glass samples. Similar to *S. aureus*, the percentage of dead *P. aeruginosa* (red-staining cells) did not increase for any of the modified samples (not shown). Representative micrographs of *P. aeruginosa* adhesion on APTES, DHP acid-1, and *p*-acid DHP are shown in Figure 4D–F.

### 2.5. Quorum Sensing Inhibition Assay

The QS inhibitory activity of the free DHP compounds was determined using *P. aeruginosa* MH602 *lasB* reporter strain (P*_lasB_*:*gfp*(ASV)) by following the protocol developed by Hentzer et al. [31]. The QS monitor strain carries a plasmid containing a green fluorescent protein (GFP) reporter gene (*gfp*(ASV)) [32] fused to the promoter of the QS-regulated *lasB* gene from *P. aeruginosa*, which causes the bacteria to produce GFP in response to QS signaling. In cell and molecular biology, the GFP gene is frequently used as a reporter of gene expression [33,34]. The short half-life of GFP (ASV) allows for repeated measurements of the same cells to continually assess gene activity over a period of time [35]. The level of GFP expression thus reflects the level of QS in the bacterial culture, and compounds that inhibit QS should result in a lowered expression of GFP. The results of the QS inhibition assay of the DHP compounds are presented in Table 4.

The *p*-acid DHP showed highest inhibition of 68.6% at 250 µM followed by DHP acid-3 and DHP acid-1 which showed 57.5% and 56.3% inhibition. At 125 µM concentration DHP acid-1 (40.4%) and p-acid DHP (38.5%) had maximum QS inhibition while the remaining DHPs displayed similar level of inhibition. The inhibition values did not vary significantly for halogenated DHPs (DHP acid 2–4) at all concentrations (62.5–250 µM). Overall, *p*-acid DHP and DHP acid-3 displayed maximum inhibition.

## 3. Discussion

In the current study, the antibacterial efficacy of DHPs attached from two points on the molecule to glass surfaces was investigated. The DHP were successfully functionalized to yield DHP acids 1–4 and p-acid DHP. The XPS analysis of the coatings demonstrated that DHPs were successfully incorporated onto the APTES-glass surfaces via either the C-4 phenyl ring (DHP acids 1–4) or the *N*-atom of the DHP (p-acid DHP). Analysis of the halogen content indicated that DHP acid-4 had the highest attachment efficiency, followed by two fluorinated DHP acid-2 and DHP acid-3. The DHP surface coatings were tested for their ability to reduce the adherent bacterial cells of *S. aureus* and *P. aeruginosa*, while the free DHPs were also tested for their QS inhibitory activity.

All DHP coatings were shown to reduce *S. aureus* and *P. aeruginosa* adhesion to the surface. For *P. aeruginosa* the orientation of the DHP does not seem to affect the activity, with all surfaces, except DHP acid 3, having similar activities. This is unexpected as in solution, the lactam ring of the DHP is known to be the more potent part of the molecule for QS inhibition than the phenyl group. This was further confirmed using the QS inhibitory activity assay, which showed that *p*-acid DHP compound (68.6% QS inhibition at 250 μM) which is unmodified at the N1-atom, was more active against *P. aeruginosa* MH602 QS than DHP acid 1–4 (<57.5%). However, this QS inhibitory effects could possibly be negated when the *p*-acid DHP is constricted when bound, as shown by our results that no general trend in the *P. aeruginosa* activity could be established between *p*-acid DHP and DHP acid 1–4.

Despite this, surfaces with DHP acid-3 with a para-fluoro on the phenyl ring showed the lowest *P. aeruginosa* adhesion, while the surfaces with a related ortho-fluoro DHP (DHP acid-2) had significantly higher adhesion in comparison. These results were consistent with a previous study by Ho et al. where analogues of DHPs were functionalized with an acrylate group at the N-1 position and grafted onto APTES glass via Michael addition reaction [26]. In that study, the activity of two fluorinated DHP acids was also different, where the para-fluoro DHP (DHP acid-3) exhibited higher activity even at low surface concentration against *P. aeruginosa* compared to ortho-fluoro compound (DHP acid-2).

For *S. aureus* activity, it was found that attachment via N-atom of the DHP resulted in lower adhesion, with 3 out of 4 surfaces (DHP acid-1, DHP acid-2, and DHP acid-4) having improved activity when compared to *p*-acid DHP attached via the C4 phenyl ring. In comparison, a previous study where the DHPs attached non-specifically via azide chemistry exhibited higher reduction in bacterial adhesion at a lower surface concentration. For instance, the *para*-bromo DHP compound exhibited low surface attachment (0.35% Br, XPS) on the azide surface but displayed bacterial reduction of 93.4% against *S. aureus* [24], compared to the specific attachment of the same *para*-bromo DHP (DHP acid-4) in this study, which had nearly three-fold higher surface attachment (0.91% Br, XPS) but exhibited only 74.2% reduction of *S. aureus* adhesion. This indicates that the choice of attachment methodology used, which affects not only the orientation but also concentration of the DHP on the surface, could have a significant influence on antibacterial efficacy.

The surface attached DHPs could act via different pathways for Gram-positive and Gram-negative bacteria. In our previous studies, DHP-coated substrates were shown to be able to disrupt the QS of *P. aeruginosa* by interference with the *N*-acylhomoserine lactones (AHL)-mediated las QS system [22]. It is possible that surface attached DHPs interact with membrane-associated receptor proteins of bacterial cells, preventing the binding of native signal and ultimately resulting in inhibition of expression of the virulence gene lasB. The current study also supports the hypothesis that surface-coated DHP interacts with membranes of bacterial cells, causing a further cascade of events, possibly including dissipation of the bacterial membrane potential that leads to reduction in cell division [36]. In addition, for *S. aureus*, exposure to AHLs have been shown to interfere with the *S. aureus agr* QS system through interaction with a specific saturable receptor on the cytoplasmic membrane. Furthermore, a derivative of AHL, 3-(1-hydroxydecylidene)-5-(2-hydroxyethyl)pyrrolidine-2,4-dione, has been shown to dissipate both the membrane potential and the pH gradient of Gram-positive bacteria including *Bacillus cereus* and *S. aureus*, resulting in cell death [37]. Therefore, interaction of surface attached DHP with *S. aureus* through at least two membrane-associated mechanisms are possible.

The physiochemical properties of the surface are also known to have a strong influence on the rate and extent of bacterial adhesion [38,39,40]. Research has shown that bacteria adhere more easily to non-polar surfaces than to hydrophilic substrates [41,42], that is, higher surface hydrophobicity results in higher biofilm development [43,44]. After amine functionalization using APTES silanization on blank glass, the hydrophobicity of the surfaces increased drastically. However, the hydrophobic APTES surface showed similar bacterial coverage as the blank glass, in agreement to previous studies [45,46]. Meanwhile, adhesion was significantly reduced after DHP attachment, even though the DHP surfaces had similar hydrophilicity (68–75° water contact angle) to the APTES surface (72°). This result implies that the reduction in bacterial colonization was caused by the DHP compounds rather than the changes in hydrophobicity of the surface.

## 4. Materials and Methods

### 4.1. General

All chemical reagents were purchased from commercial sources (Alfa-Aesar and Sigma Aldrich, Sydney, Australia) and used without further purification. Solvents were sourced from commercial sources and used as obtained. Reactions were performed using oven-dried glassware under an atmosphere of nitrogen and in anhydrous conditions (as required). Room temperature (rt) refers to the ambient temperature (22–24 °C). Yields refer to chromatographically and spectroscopically pure compounds unless otherwise stated. Reactions were monitored by thin layer chromatography (TLC) precoated with Merck silica gel 60 F254 (Sigma Aldrich, Sydney, Australia). Visualization was performed by the quenching of short or long wavelength UV fluorescence or by staining with potassium permanganate or ninhydrin solution. Flash chromatography was carried out using Grace Davison LC60A 6–35 µm silica gel (Discovery Sciences, Epping, Australia). Preparative thin layer chromatography was carried out on 3 × 200 × 200 mm glass plates coated with Merck 60GF_254_ silica gel. Infrared spectra were recorded using a Cary 630 FTIR spectrophotometer (Agilent Technologies Australia, Mulgrave, Australia). Ultraviolet spectra were measured using a Cary 100 Bio UV-visible spectrophotometer (Agilent Technologies Australia, Mulgrave, Australia) in the designated solvents and data reported as wavelength (λ) in nm and absorption coefficient (ε) in cm^−1^ M^−1^. High resolution mass spectrometry was performed by the Bioanalytical Mass Spectrometry facility, UNSW. Melting points were obtained using Mel-Temp melting point apparatus and are uncorrected. Proton and carbon NMR was recorded in designated solvents using Bruker DPX 300 or a Bruker Avance 400 spectrometer (Bruker Pty Ltd, Preston, Australia) as designated. Chemical shifts (δ) are quoted in parts per million (ppm), to the nearest 0.01 ppm and internally referenced relative to the solvent nuclei. ^1^H-NMR spectral data are reported as follows: [chemical shift in ppm; multiplicity in br, broad; s, singlet; d, doublet; t, triplet; q, quartet; quint, quintet; sext, sextet; sept, septet; m, multiplet; or as a combination of these (e.g. dd, dt etc.)]; coupling constant (*J*) in hertz, integration, proton count, and assignment.

### 4.2. Synthesis of DHP Derivatives

#### 4.2.1. *Tert*-Butyl-2-(5-(2-(*tert*-butoxy)-2-oxoethoxy)-5-methyl-2-oxo-4-phenyl-1,5-dihydro-2*H*-pyrrol-1-yl) acetate (3, R = H)

5-Hydroxy-5-methyl-4-phenyl-1,5-dihydro-2*H*-pyrrol-2-one (1.0 g, 2.58 mmol) was added to a solution of potassium hydroxide (0.6 g, 10.57 mmol) in dry DMSO (5 mL) and the mixture was stirred at room temperature for 20 min. To the solution *tert*-butyl chloroacetate (1.6 g, 10.57 mmol) was added and stirred at room temperature for 24 h. The crude mixture was washed with water and extracted into ethyl acetate. The extracted organic layer was dried over sodium sulphate, evaporated under vacuum and flash columned to yield a white solid (1.36 g, 61%). M.p. 128 °C; ^1^H-NMR (300 MHz, CDCl_3_) δ 1.46 (d, *J* = 8.6 Hz, 21H, 7 × CH_3_), 4.01 (s, 2H, CH_2_), 4.34 (s, 2H, CH_2_), 6.26 (d, *J* = 1.2 Hz, 1H, CH), 7.44 (s, 5H, ArH); ^13^C-NMR (100 MHz, CDCl_3_) δ 23.2 (CH_3_), 28.0 (6 × CH_3_), 41.6 (CH_2_), 60.9 (CH_2_), 82.4 (2 × C-O), 96.9 (CH), 121.0 (C), 128.6 (2 × ArCH), 129.4 (ArCH), 131.9 (2 × ArCH), 145.1 (ArC), 150.7 (C), 167.2 (C=O), 168.9 (C=O), 172.6 (C=O); IR (ATR): υ_max_ 3071, 2972, 1725, 1703, 1389, 1227, 1150, 1084, 916, 837, 769, 695 cm^−1^; UV (ACN): λ_max_ 207 nm (ε 8970 cm^−1^M^−1^), 218 (9762), 261 (11,848); HRMS (ESI) *m/z* calcd for C_23_H_31_NO_6_Na 440.2044 [M + Na]^+^, found 440.2042.

#### 4.2.2. 2-(5-Methylene-2-oxo-4-phenyl-1,5-dihydro-2*H*-pyrrol-1-yl)acetic acid (DHP acid-1)

Trifluoroacetic acid (2.5 mL) was added to a solution of *tert*-butyl-2-(2-(2-(*tert*-butoxy)-2-oxoethoxy)-2-methyl-5-oxo-3-phenyl-2,5-dihydro-1H-pyrrol-1-yl) acetate (1.1 g, 2.76 mmol) in dichloromethane (15 mL) and the mixture was stirred at room temperature for 24 h. The reaction mixture was washed with saturated sodium bicarbonate and water. The ethyl acetate layer was separated, dried over sodium sulphate and chromatographed to yield a white solid (0.38 g, 69%). M.p. 165–166 °C; ^1^H-NMR (300 MHz, CDCl_3_) δ 4.52 (s, 2H, CH_2_), 4.98 (dd, *J* = 2.7 and 1.7 Hz, 1H, =CH_2_), 5.08 (d, *J* = 2.7 Hz, 1H, =CH_2_), 6.30 (s, 1H, CH), 7.47 (s, 5H, ArH); ^13^C-NMR (100 MHz, CDCl_3_) δ 40.5 (CH_2_), 97.4 (CH_2_), 120.8 (CH), 128.6 (4 × ArCH), 129.6 (ArCH), 131.6 (ArC), 144.7 (C), 151.6 (C), 171.4 (C=O), 175.0 (C=O); IR (ATR): υ_max_ 2905, 2828, 2725, 2601, 2533, 1732, 1650, 1625, 1431, 1341, 1211, 1146, 864, 765, 704, 700 cm^−1^; UV (ACN): λ_max_ 276 nm (ε 13,011 cm^−1^ M^−1^); HRMS (ESI) *m/z* calcd for C_13_H_11_NO_3_Na 252.0631 [M + Na]^+^, found 252.0632.

Other derivatives (DHP acid 2–4) were synthesized by following the same method from the corresponding *tert*-butyl acetate DHPs.

#### 4.2.3. 2-(5-Methylene-2-oxo-4-(2-fluorophenyl)-1,5-dihydro-2*H*-pyrrol-1-yl)acetic acid (DHP acid-2)

M.p. 150 °C; ^1^H-NMR (400 MHz, CDCl_3_) δ 4.51 (s, 2H, CH_2_), 4.94–4.97 (m, 2H, =CH_2_), 6.39 (s, 1H, CH), 7.16–7.24 (m, 2H, ArH), 7.35–7.45 (m, 2H, ArH); ^13^C-NMR (100 MHz, CDCl_3_) δ 40.5 (CH_2_), 97.1 (CH_2_), 116.2 (CH), 116.4 (ArCH), 123.5 (ArC), 124.2 (ArCH), 130.9 (ArCH), 131.3 (ArCH), 144.5 (C), 145.0 (C), 161.0 (ArCF), 169.0 (C=O), 171.5 (C=O); IR (ATR): υ_max_ 1549, 2916, 2603, 2537, 2104, 1931, 1727, 1624, 1486, 1432, 1352, 1208, 1085, 882, 832, 759 cm^−1^; UV (ACN): λ_max_ 272 nm (ε 10,352 cm^−1^ M^−1^); HRMS (ESI) *m/z* calcd for C_13_H_10_FNO_3_Na 270.0537 [M + Na]^+^, found 270.0535.

#### 4.2.4. 2-(5-Methylene-2-oxo-4-(4-fluorophenyl)-1,5-dihydro-2*H*-pyrrol-1-yl)acetic acid (DHP acid-3)

M.p. 201–202 °C; ^1^H-NMR (400 MHz, CDCl_3_) δ 4.50 (s, 2H, CH_2_), 4.96 (t, *J* = 2.7 Hz, 1H, =CH_2_), 5.01 (d, *J* = 2.7 Hz, 1H, =CH_2_), 6.26 (s, 1H, CH), 7.13–7.17 (m, 2H, ArH), 7.41–7.44 (m, 2H, ArH); ^13^C-NMR (100 MHz, CDCl_3_) δ 40.4 (CH_2_), 97.2 (CH_2_), 115.8 (CH), 116.1 (2 × ArCH), 120.9 (ArC), 130.4 (2 × ArCH), 144.7 (C), 150.0 (C), 162.0 (ArCF), 168.9 (C=O), 172.0 (C=O); IR (ATR): υ_max_ 2886, 2729, 2606, 2538, 2116, 1733, 1655, 1628, 1501, 1430, 1351, 1212, 1144, 916, 882, 845, 770 cm^−1^; HRMS (ESI) *m/z* calcd for C_13_H_10_FNO_3_Na 270.0537 [M + Na]^+^, found 270.0534.

#### 4.2.5. 2-(5-Methylene-2-oxo-4-(4-bromophenyl)-1,5-dihydro-2*H*-pyrrol-1-yl)acetic acid (DHP acid-4)

M.p. 191 °C; ^1^H-NMR (300 MHz, Acetone-d_6_) δ 4.49 (s, 2H, CH_2_), 4.04 (d, *J* = 2.4 Hz, 1H, =CH_2_), 5.19 (s, 1H, =CH_2_), 6.4 (s, 1H, CH), 7.51 (dd, *J* = 58.2 and 8.4 Hz); ^13^C-NMR (75.5 MHz, Acetone-d_6_) δ 40.0 (CH_2_), 96.1 (CH_2_), 121.3 (CH), 123.2 (ArCBr), 130.5 (2 × ArCH), 131.5 (C), 132.0 (2 × ArCH), 144.7 (ArC), 149.3 (C), 167.9 (C=O), 168.7 (C=O); IR (ATR): υ_max_ 3350, 2918, 2733, 2528, 2110, 1908, 1719, 1664, 1483, 1433, 1396, 1349, 1217, 1199, 1069, 873, 824 cm^−1^; UV (ACN): λ_max_ 222 nm (ε 10,897 cm^−1^ M^−1^), 278 (11,112); HRMS (ESI) *m/z* calcd for C_13_H_10_BrNO_3_Na 329.9736 [M + Na]^+^, found 329.9735.

#### 4.2.6. (*Z*)-4-(1-Carboxy-3-oxobut-1-en-2-yl)benzoic acid **7**

Phosphoric acid (15 mL) was added to a solution of 4-(2-oxopropyl)benzoic acid (0.35 g, 1.5 mmol) and glyoxylic acid (0.37 g, 4 mmol) and the mixture was heated at 75–80 °C for 5 h. The mixture was cooled to room temperature, extracted into 1:1 DCM/ether layer and dried over MgSO_4_. The solvent was evaporated under vacuum and purified by flash chromatography with 5:1 ethyl acetate/methanol to obtain a pale yellow solid (0.18 g, 50%). M.p. 114–116 °C; ^1^H-NMR (300 MHz, DMSO-*d*_6_) δ 1.68 (s, 3H, CH_3_), δ 6.8 (s, 1H, =CH), δ 8.0–8.05 (m, 4H, ArH), δ 13.1 (brs, 1H, COOH).

#### 4.2.7. 5-Hydroxy-5-methyl-4-(4-carboxyphenyl)-1,5-dihydro-2*H*-pyrrol-2-one **10**

(*Z*)-4-(1-Carboxy-3-oxobut-1-en-2-yl)benzoic acid was treated with trifluoroacetic acid (5 mL) at room temperature for 1 h. The solvent was removed under vacuum to yield a brown solid, presumably 5-hydroxy-5-methyl-4-(4-carboxyphenyl)-2(5*H*)furanone (0.1 g, 0.42 mmol), which was dissolved in thionyl chloride (5 mL) and stirred at room temperature for 24 h. The excess thionyl chloride was removed from the reaction mixture under high vacuum. The residue was stirred with ice-cold water for few minutes and then with aqueous ammonia (7 mL, 30%) for 3 h. The excess ammonia was removed in vacuo and the residue was acidified with 2 M HCl (3 mL) to obtain a brown precipitate which was filtered under vacuum and subjected to flash chromatography with EtOAc/MeOH (9:1) to yield the title product as a white solid (0.035 g, 35%). M.p. 198–200 °C; ^1^H-NMR (300 MHz, DMSO-*d*_6_) δ 1.56 (s, 3H, CH_3_), 3.7 (brs, 1H, OH), 6.7 (s, 1H, CH), 7.5 (s, 1H, ArH), 7.9 (m, 3H, ArH), 8.5 (s, 1H, NH), 13.0 (brs, 1H, COOH); ^13^C-NMR (75.5 MHz,DMSO-*d*_6_) δ 26.7 (CH_3_), 107.0 (C), 117.0 (CH), 128.9 (2 × ArCH), 130.2 (ArC), 130.5 (2 × ArCH), 133.6 (ArC), 164.5 (C), 168.0 (C=O), 170.0 (C=O); HRMS (ESI) *m/z* calcd for C_12_H_11_NO_4_Na 256.0580 [M + Na]^+^, found 256.0580.

#### 4.2.8. 4-(4-Carboxy phenyl)-5-methyelene-1,5-dihydro-2*H*-pyrrol-2-one (*p*-acid DHP) **11**

5-hydroxy-5-methyl-4-(4-carboxyphenyl)-1,5-dihydro-2*H*-pyrrol-2-one (0.2 g, 0.85 mmol) in borontrifluoride dietherate (2 mL) was stirred at room temperature for 24 h. The resulting reaction mixture was filtered under vacuum, washed with cold water and dried. The residue was chromatographed on silica gel using EtOAc/MeOH (5:1) to yield the final product as a pale yellow solid (0.10 g, 50%). M.p. 145–146 °C; ^1^H-NMR (300 MHz, DMSO-*d*_6_) δ 4.91 (s, 1H, CH), 5.19 (s, 1H, =CH_2_), 6.42 (s, 1H, =CH_2_), 7.46–7.59 (m, 2H, ArH), 7.95–8.08 (m, 2H, ArH), 10.2 (1H, COOH); ^13^C-NMR (75.5 MHz,DMSO-*d*_6_) δ 98.0 (CH_2_), 123.7 (CH), 128.3 (2 × ArCH), 128.8 (2 × ArCH), 134.6 (ArC), 135.3 (C), 144.1 (ArC), 149.3 (C), 167.7 (C=O), 170.1 (C=O); IR (ATR): υ_max_ 3457, 3361, 3200, 2358, 2257, 2109, 1634, 1600, 1409, 1189, 1033, 924, 848, 770, 741 cm^−1^; UV (THF): λ_max_ 236 nm (ε 8817 cm^−1^ M^−1^), 281 (3935); HRMS (ESI) *m/z* calcd for C_12_H_9_NO_3_H 216.0655 [M + H]^+^, found 216.0655.

### 4.3. Attachment of (3-Aminopropyl)triethoxysilane (APTES)

Glass coverslips (No. 1, diameter 13 mm D 263 M glass, ProSciTech Pty Ltd, Kirwan Australia) were first cleaned in freshly prepared piranha solution (3:1 *v/v* concentrated sulphuric acid to 30% hydrogen peroxide) at 100 °C for 1 h. After thorough rinsing with distilled water, the clean coverslips were rinsed once with absolute ethanol and air-dried.

The coverslips were then silanized according to the previously developed method [26]. Briefly, the clean substrates were placed on a steel mesh within a glass vessel that contains a (3-aminopropyl)triethoxysilane (APTES) solution (10% *v/v* in dry toluene; 1 mL). The glass vessel was sealed and heated at 140 °C for 18 h. The coverslips were rinsed with dry toluene (×2), absolute ethanol and air-dried.

### 4.4. Attachment of DHPs

A solution containing DHP (20.2 µM), EDC (101.2 µM), NHS (40.5 µM) and a small crystal of 4-dimethylaminopyridine (DMAP) in 1:1 ethanol/water was prepared. The amine-functionalized APTES glass surface was immersed in 1.5 mL of this solution and agitated overnight. The unreacted DHP was removed by extensively washing the samples with MilliQ water and absolute ethanol, and the surfaces were then air dried and stored in a clean sterile container.

### 4.5. X-ray Photoelectron Spectroscopy

X-ray photoelectron spectroscopy measurements were performed on an ESCALAB 220iXL Monochromatic Al Kα X-rays (1486.6 eV) (ThermoFisher Scientific, North Ryde, Australia) incident at 58° to the analyzer lens were used to excite electrons from the sample. Emitted photoelectrons were collected on a hemispherical analyzer with a multichannel detector at a take-off angle of 90° from the plane of the sample surface. The analyzing chamber operated below 10–8 Torr, and the spot size was approximately 1 mm^2^. The resolution of the spectrometer was ~0.6 eV. All energies are reported as binding energies in eV and referenced to the C 1s signal (corrected to 285.0 eV). Survey scans were carried out at 100 ms dwell time and an analyzer pass energy of 100 eV. High-resolution scans were run with 0.1 eV step size, dwell time of 100 ms, and analyzer pass energy set to 20 eV. After background subtraction using the Shirley routine, spectra were fitted with a convolution of Lorentzian and Gaussian profiles as described by Ciampi et al. [47].

### 4.6. Contact Angle Measurements

Contact angles were determined using a contact angle goniometer (Rame-Hart, Inc, Succasunna, NJ, USA, Model no. 100-00). Multiple drops of deionized water were placed on each surface using a micro-syringe. The angle between the droplet and the surface was measured using a 50 mm Cosmicar Television Lens (Japan). Rame-Hart Imaging software (2002) was used to calculate the contact angle. A minimum of fifteen measurements were made on five samples.

### 4.7. Bacterial Adhesion Analysis

The bacterial strains used for this study, *Staphylococcus aureus* SA38 and *Pseudomonas aeruginosa* PA01, were streaked on chocolate agar (Oxoid, ThermoFisher Scientific, North Ryde, Australia) and incubated at 37 °C overnight. A single colony of the bacteria from the pate was cultured overnight in 15 mL tryptone soya broth (TSB; Oxoid, ThermoFisher Scientific, North Ryde, Australia) medium at 37 °C. The bacteria were washed twice with the same volume of fresh TSB by centrifugation. The optical density of the resulting culture was adjusted to OD_660_ = 0.1 in TSB which corresponds to 1 × 10^8^ cfu mL^−1^.

The surfaces to be tested were first sterilized with 70% *w/v* ethanol for 30 min in a 12-well plate, then thoroughly washed with phosphate buffered saline (PBS) three times and finally placed in 4 mL of the above bacterial suspension. The plates were incubated at 37 °C, after 24 h the bacterial suspension was replaced with 4 mL of fresh TSB and incubated as before for the next 24 h. The samples were gently washed twice with PBS to remove non-adherent bacteria before examining for bacterial adhesion by florescence microscopy.

Surfaces with adherent bacterial cells prepared as described above were stained with the Live/Dead BacLight Bacterial Viability Kit L-7007 (Molecular Probes Inc, Eugene, OR, USA) according to the manufacturers’ procedure and as described in the literature for analysis of biofilms on surfaces [22,26,48,49]. Briefly, 2 µL of the two components were mixed thoroughly in 1 mL of PBS; 100 µL of the solution was then trapped between the sample and the glass microscopy slide and allowed to incubate at room temperature in the dark for 15 min. Bacteria were then fixed by adding 100 µL of 4% formaldehyde to each sample.

Microscopic observation and image acquisition were performed with an Olympus FV1200 Confocal Inverted Microscope (Olympus Australia Pty Ltd, Notting Hill, Australia). The bacterial cells that were stained green were considered to be viable, while those that stained red or both green and red were considered to be dead. Images from 10 representative areas on each of triplicate samples for each surface were taken and analyzed using ImageJ software [50]. The results were reported as the average percentage coverage of live and dead cells of the fields of view.

### 4.8. Statistical Analysis of Data

For bacterial attachment assay further analysis of the was done by the one-way analysis of variance (ANOVA) using GraphPad Prism 7.03 software (GraphPad Software Inc, San Diego, CA, USA). Post hoc multiple comparisons were done using Tukey correction.

### 4.9. Quorum Sensing Inhibition Assay

To evaluate the effectiveness of the synthesized DHP derivatives against QS signaling, the *P. aeruginosa* MH602 P*_lasB_::gfp* (ASV) reporter strain, which harbors a chromosomal fusion of the *lasB* promoter to an unstable gfp gene and which responds to the AHL 3-oxo-dodecanoyl homoserine lactone (3oxo-C12-HSL), was used [31]. To each well of the top row in a 96-well plate, 160 μL of Luria–Bertani (LB_10_) broth medium and 40 μL of 5 mM test compound in DMSO were added. The test compound was diluted by two-fold each time in LB_10_ broth medium in all subsequent wells. Then, 100 μL of a 100-times diluted overnight culture of *P. aeruginosa* MH602 in LB_10_ broth was added to all wells, and the final volume in each well was 200 μL. The plates were incubated at 37 °C for 15 h in a microplate reader (Wallac Victor, Perkin-Elmer, Melbourne, Australia), and every 30 min the plates were briefly shaken and measured for GFP expression (fluorescence: excitation 485 nm, emission 535 nm) and cell growth (OD_600_). The inhibitory effect of a DMSO control (1% of total volume) was examined in similar fashion but no inhibitory effect on either GFP expression or cell growth was observed.

## 5. Conclusions

In this study, the effect of molecular orientation of surface-immobilized DHPs on biological activity was evaluated by attaching different parts of the DHP to the surface using a specific attachment strategy. The *N*-substituted acid DHPs 1–4 and the *p*-acid DHP were effective at reducing bacterial colonization of *S. aureus* and *P. aeruginosa* on glass surfaces. This study demonstrated the importance of the orientation of the DHP for *S. aureus* activity where the best activity occurred when the DHP was attached to the surface via N-1 position of the lactam ring, exposing the pendant phenyl ring. While for *P. aeruginosa* activity, no difference was observed between the two attachment strategies. Future SAR studies on the antimicrobial activity of different substituents on the phenyl ring of attached DHPs will be carried out to further explore the difference in activity against Gram-positive and Gram-negative bacteria.

## Figures and Tables

**Table 1:** XPS elemental composition of blank, (3-Aminopropyl)triethoxysilane (APTES) and DHP-modified glass surfaces.

Surface	%C	%N	%Halogen
Blank	6.6	0.6	-
APTES	45.4	8.1	-
DHP acid-1	48.6	8.9	-
DHP acid-2	49.3	8.8	0.40% F
DHP acid-3	47.2	10.8	0.17% F
DHP acid-4	51.7	10.3	0.91% Br
*p*-acid DHP	50.2	9.2	-

**Table 2:** XPS binding energies for C1s and N1s and proposed assignments with percentage peak intensities.

Surface	C 1s			N 1s		
	Binding energy (eV)	Assignment	Peak area (%)	Binding energy (eV)	Assignment	Peak area (%)
**APTES**	284.9	C-C	63.0	399.5	NH_2_	80.2
	285.9	C-N	25.5	401.4	NH_3_^+^, Tertiary N	19.7
	287.9	C=O	11.4			
**DHP acid-1**	284.8	C-C	57.0	399.5	NH_2_	34.0
	285.8	C-N	21.0	400.2	N-C=O	56.6
	287.8	C=O	11.6	401.6	NH_3_^+^, Tertiary N	9.4
	288.6	N-C=O	10.4			
**DHP acid-2**	284.8	C-C	56.7	399.5	NH_2_	30.9
	285.8	C-N	21.2	400.2	N-C=O	58.3
	287.8	C=O	12.1	401.6	NH_3_^+^, Tertiary N	10.8
	288.6	N-C=O	10.0			
**DHP acid-3**	284.8	C-C	55.8	399.4	NH_2_	21.7
	285.8	C-N	24.2	400.0	N-C=O	67.6
	287.8	C=O	10.0	401.6	NH_3_^+^, Tertiary N	10.7
	288.7	N-C=O	10.0			
**DHP acid-4**	284.8	C-C	60.0	399.4	NH_2_	36.8
	285.8	C-N	22.8	400.0	N-C=O	59.7
	287.6	C=O	8.8	401.4	NH_3_^+^, Tertiary N	3.5
	288.6	N-C=O	8.4			
*p* **-acid DHP**	284.8	C-C	67.2	399.6	NH_2_	61.6
	285.8	C-N	12.0	400.1	N-C=O	31.3
	287.9	C=O	14.1	401.5	NH_3_^+^, Tertiary N	7.1
	288.4	N-C=O	6.6			

**Table 3:** Contact angle measurements of glass substrates.

Surface	Contact Angle (°) (±1)
Blank	19
APTES	72
DHP acid-1	71
DHP acid-2	73
DHP acid-3	74
DHP acid-4	75
*p*-acid DHP	68

**Table 4:** QS inhibition activity (%) against *P. aeruginosa* MH602 lasB reporter strain at different concentrations of DHP derivatives; ^a^growth inhibition ≤10%; ^b^growth inhibition 11%–30%; growth inhibition >30%.

Compound	QS Inhibition against PA MH602 (%)		
	62.5 µM	125 µM	250 µM
DHP acid-1	27.6 ± 1.7^a^	40.4 ± 1.2^a^	56.3 ± 0.4^b^
DHP acid-2	33.5 ± 1.2^a^	34.2 ± 2.3^a^	55.3 ± 3.8^a^
DHP acid-3	33.0 ± 1.8^a^	35.2 ± 2.0^a^	57.5 ± 0.7^b^
DHP acid-4	31.0 ± 1.7^a^	35.1 ± 1.1^a^	53.6 ± 1.5^a^
p-acid DHP	35.0 ± 2.4^a^	38.5 ± 1.1^b^	68.6 ± 1.1^b^
Furanone-30 (positive control)	41.4 ± 2.7^a^	64.2 ± 3.5^b^	89.7 ± 4.6^c^
